# APP Gene Duplication Unraveled: A Unique Case of Atypical Dementia with Prominent Psychiatric Concerns in a Hispanic Male

**DOI:** 10.1002/alz.087107

**Published:** 2025-01-03

**Authors:** Julia Castro, Sandra Azareli Garcia Velazquez, Tiffany F. Kautz, Crystal Mendoza, Arash Salardini, Sudha Seshadri, Alicia S. Parker, A. Campbell Sullivan

**Affiliations:** ^1^ Glenn Biggs Institute for Alzheimer’s and Neurodegenerative Diseases, University of Texas Health San Antonio, San Antonio, TX USA; ^2^ Department of Neurology, University of Texas Health San Antonio, San Antonio, TX USA; ^3^ Glenn Biggs Institute for Alzheimer’s & Neurodegenerative Diseases, University of Texas Health Science Center, San Antonio, TX USA; ^4^ South Texas Alzheimer’s Disease Research Center, San Antonio, TX USA; ^5^ Glenn Biggs Institute for Alzheimer’s & Neurodegenerative Diseases, San Antonio, TX USA; ^6^ Division of Neuropsychology, University of Texas Health Science Center at San Antonio, San Antonio, TX USA

## Abstract

**Background:**

APP duplications are a rare form of familial Alzheimer’s disease (AD). Research has shown variability in clinical presentation with full duplications. There is limited information on those with partial duplications, especially in underrepresented minorities.

**Methods:**

A 64 y/o Hispanic male presented with a 2.5 year history of anxiety, restlessness, coarsening behavior, cognitive and functional decline, and motor changes. We present findings from neuropsychological evaluation, neurological exam, neuroimaging, and genetic testing. Plasma‐based biomarkers are pending.

**Results:**

The clinical course is notable for rapid onset of anxiety at age 62 with intrusive thoughts, delusional beliefs, and repeated psychiatric hospitalizations. Motor changes include myoclonic jerks, increased restless with pacing and rocking, and bradykinesia. The family described a 30lb weight loss without dieting, dietary changes with a preference for sweets, withdrawal from social interactions, and poor judgment. DaT scan was positive; synuclein biopsy was negative. Brain MRI revealed remote left basal ganglia lacune and superficial parietal atrophy. Neuropsychological assessment revealed prominent executive dysfunction, and amnestic verbal learning and recall, with preserved visual learning. Family history included late‐onset forgetfulness in his mother and abnormal behaviors in maternal half‐cousins.

Genetic sequencing uncovered a partial APP gene duplication (exons 1‐11, including the initiator codon). Illumina SNP chromosomal microarray confirmed a 21q duplication also including the CYYR‐1 and CYYR1‐AS1 genes, with the partial APP duplication defined as “uncertain” per ACMG guidelines.

**Conclusion:**

This case study describes an atypical dementia syndrome in a 64 y/o Hispanic male with a partial APP gene duplication. The clinical syndrome is notable for neuropsychiatric symptoms and parkinsonism. This report is consistent with recent work highlighting atypical dementia among a small proportion of those with APP duplications. However, our findings show the ramifications of a partial duplication within an underrepresented population, emphasizing the need for further exploration.

